# Supplementary material for: Meta-Analysis of Steel Fiber-Reinforced Concrete Mixtures Leads to Practical Mix Design Methodology
Source: Materials (Basel). 2021 Jul 13;14(14):3900. doi: 10.3390/ma14143900 (PMC8307720; doi:10.3390/ma14143900)
Supplement: Supplementary file 1 [file materials-14-03900-s001.zip › materials-1283883-supplementary.pdf]

Supplemental File

Meta-Analysis of Steel Fiber-Reinforced Concrete Mixtures Leads to Practical Mix Design Methodology

Emilio Garcia-Taengua [1,*], Mehdi Bakhshi [2] and Liberato Ferrara [3]

[1] School of Civil Engineering, University of Leeds, LS2 9JT Leeds, UK
[2] AECOM, Los Angeles, CA 90071, USA; Mehdi.Bakhshi@aecom.com
[3] Department of Civil and Environmental Engineering, Politecnico di Milano, 20133 Milan, Italy; liberato.ferrara@polimi.it
* Correspondence: E.Garcia-Taengua@leeds.ac.uk

DATABASE AND VALIDATION DATASET –LIST OF SOURCES

Abdallah, Sadoon, Mizi Fan, Xiangming Zhou, and Simon Le Geyt. "Anchorage effects of various steel fibre architectures for concrete reinforcement." International Journal of Concrete Structures and Materials 10, no. 3 (2016): 325-335.

Ding, Yining. "Investigations into the relationship between deflection and crack mouth opening displacement of SFRC beam." Construction and Building Materials 25, no. 5 (2011): 2432-2440.

Lee, Jong-Han, Baiksoon Cho, and Eunsoo Choi. "Flexural capacity of fiber reinforced concrete with a consideration of concrete strength and fiber content." Construction and Building Materials 138 (2017): 222-231.

Bosman, Tiaan, and Elsabe P. Kearsley. "The influence of fibre spatial characteristics on the flexural performance of SFRC." Materials and Structures 52, no. 3 (2019): 51.

Zhang, Shengli, Changsuo Zhang, and Lin Liao. "Investigation on the relationship between the steel fibre distribution and the post-cracking behaviour of SFRC." Construction and Building Materials 200 (2019): 539-550.

Michels, Julien, Rouven Christen, and Danièle Waldmann. "Experimental and numerical investigation on postcracking behavior of steel fiber reinforced concrete." Engineering Fracture Mechanics 98 (2013): 326-349.

Paegle, Ieva, Fausto Minelli, and Gregor Fischer. "Cracking and load-deformation behavior of fiber reinforced concrete: Influence of testing method." Cement and Concrete Composites 73 (2016): 147-163.

Casas Tuneu, Joan. "Assessment of fibre distribution and orientation within SFRC precast segments." Bachelor's thesis, Universitat Politècnica de Catalunya, 2015.

Salehian, Hamidreza, and Joaquim AO Barros. "Assessment of the performance of steel fibre reinforced self-compacting concrete in elevated slabs." Cement and Concrete Composites 55 (2015): 268-280.

Komarkova, T., J. Lanik, L. Topolar, J. Stoller, and P. Stonis. "Experimental assessment of steel fibre reinforced concretes with different concentrations of fibres." In IOP Conference Series: Materials Science and Engineering, vol. 385, no. 1, p. 012028. IOP Publishing, 2018.

Yoo, Doo-Yeol, Young-Soo Yoon, and Nemkumar Banthia. "Predicting the post-cracking behavior of normal-and high-strength steel-fiber-reinforced concrete beams." Construction and Building Materials 93 (2015): 477-485.

Poveda, Elisa, Gonzalo Ruiz, Hector Cifuentes, C. Yu Rena, and Xiaoxin Zhang. "Influence of the fiber content on the compressive low-cycle fatigue behavior of self-compacting SFRC." International Journal of Fatigue 101 (2017): 9-17.

Papachristoforou, Michail, and Ioanna Papayianni. "Radiation shielding and mechanical properties of steel fiber reinforced concrete (SFRC) produced with EAF slag aggregates." Radiation Physics and Chemistry 149 (2018): 26-32.

Domski, Jacek. "A blurred border between ordinary concrete and SFRC." Construction and Building Materials 112 (2016): 247-252.

di Prisco, Marco, Matteo Colombo, and Ali Pourzarabi. "Biaxial bending of SFRC slabs: Is conventional reinforcement necessary?." Materials and Structures 52, no. 1 (2019): 1.

Lim, Sopokhem, Ramiz Ahmed Raju, Mitsuhiro Matsuda, Takehiro Okamoto, and Mitsuyoshi Akiyama. "Structural behavior prediction of SFRC beams by a novel integrated approach of X-ray imaging and finite element method." Construction and Building Materials 170 (2018): 347-365.

Mahmud, Goran H., Zhenjun Yang, and Aram MT Hassan. "Experimental and numerical studies of size effects of Ultra High Performance Steel Fibre Reinforced Concrete (UHPFRC) beams." Construction and Building Materials 48 (2013): 1027-1034.

Zhang, Shengli, Lin Liao, Shuizhou Song, and Changsuo Zhang. "Experimental and analytical study of the fibre distribution in SFRC: A comparison between image processing and the inductive test." Composite Structures 188 (2018): 78-88.

Choi, Won-Chang, Kwon-Young Jung, Seok-Joon Jang, and Hyun-Do Yun. "The Influence of Steel Fiber Tensile Strengths and Aspect Ratios on the Fracture Properties of High-Strength Concrete." Materials 12, no. 13 (2019): 2105.

Lee, Jong-Han. "Influence of concrete strength combined with fiber content in the residual flexural strengths of fiber reinforced concrete." Composite Structures 168 (2017): 216-225.

Mínguez, Jesús, Dorys C. González, and Miguel A. Vicente. "Fiber geometrical parameters of fiber-reinforced high strength concrete and their influence on the residual post-peak flexural tensile strength." Construction and Building Materials 168 (2018): 906-922.

Venkateshwaran, Akshay, Kiang Hwee Tan, and Yi Li. "Residual flexural strengths of steel fiber reinforced concrete with multiple hooked-end fibers." Structural Concrete 19, no. 2 (2018): 352-365.

Bhosale, Aniket, M. Abdur Rasheed, S. Suriya Prakash, and Gangadharan Raju. "A study on the efficiency of steel vs. synthetic vs. hybrid fibers on fracture behavior of concrete in flexure using acoustic emission." Construction and Building Materials 199 (2019): 256-268.

De Smedt, Maure, Kristof De Wilder, Lucie Vandewalle, and Els Verstrynge. "Acoustic Emission-based analysis of damage mechanisms in steel fibre reinforced concrete under monotonic and cyclic loading." In Proceedings of the 10th International Conference on Fracture Mechanics of Concrete and Concrete Structures, pp. 1-11. IA-FraMCoS, 2019.

Yoo, Doo-Yeol, Soonho Kim, Gi-Joon Park, Jung-Jun Park, and Sung-Wook Kim. "Effects of fiber shape, aspect ratio, and volume fraction on flexural behavior of ultra-high-performance fiber-reinforced cement composites." Composite Structures 174 (2017): 375-388.

Facconi, Luca, Fausto Minelli, and Giovanni Plizzari. "Steel fiber reinforced self-compacting concrete thin slabs–Experimental study and verification against Model Code 2010 provisions." Engineering Structures 122 (2016): 226-237.

Pająk, Małgorzata, and Tomasz Ponikiewski. "Investigation on concrete reinforced with two types of hooked fibers under flexure." Procedia Engineering 193 (2017): 128-135.

Holschemacher, K., T. Mueller, and Y. Ribakov. "Effect of steel fibres on mechanical properties of high-strength concrete." Materials & Design (1980-2015) 31, no. 5 (2010): 2604-2615.

Gouveia, Nuno D., Nelson AG Fernandes, Duarte MV Faria, António MP Ramos, and Válter JG Lúcio. "SFRC flat slabs punching behaviour–Experimental research." Composites Part B: Engineering 63 (2014): 161-171.

Rizzuti, Lidia, and Francesco Bencardino. "Effects of fibre volume fraction on the compressive and flexural experimental behaviour of SFRC." Contemporary Engineering Sciences 7, no. 8 (2014): 379-390.

Parmentier, Benoit, Petra Van Itterbeeck, and Audrey Skowron. "The flexural behaviour of SFRC flat slabs: the Limelette full-scale experiments for supporting design model codes." In Proceedings of FRC. 2014.

Kim, Wha-Jung, M. S. Kwak, and J. C. Lee. "Fracture properties of high-strength steel fiber concrete." In Proceedings of the Korea Concrete Institute Conference. Korea Concrete Institute, 2010.

Pająk, M., and T. Ponikiewski. "Flexural behavior of self-compacting concrete reinforced with different types of steel fibers." Construction and Building materials 47 (2013): 397-408.

Bencardino, Francesco, Lidia Rizzuti, Giuseppe Spadea, and Ramnath Narayan Swamy. "Implications of test methodology on post-cracking and fracture behaviour of steel fibre reinforced concrete." Composites Part B: Engineering 46 (2013): 31-38.

Soetens, Tim, and Stijn Matthys. "Different methods to model the post-cracking behaviour of hooked-end steel fibre reinforced concrete." Construction and Building Materials 73 (2014): 458-471.

Şahin, Yuşa, and Fuat Köksal. "The influences of matrix and steel fibre tensile strengths on the fracture energy of high-strength concrete." Construction and Building Materials 25, no. 4 (2011): 1801-1806.

Soulioti, D. V., N. M. Barkoula, A. Paipetis, and T. E. Matikas. "Effects of fibre geometry and volume fraction on the flexural behaviour of steel-fibre reinforced concrete." Strain 47 (2011): e535-e541.

de Montaignac, Renaud, Bruno Massicotte, Jean-Philippe Charron, and Ali Nour. "Design of SFRC structural elements: post-cracking tensile strength measurement." Materials and Structures 45, no. 4 (2012): 609-622.

Aoude, Hassan, and Michael Cohen. "Shear response of SFRC beams constructed with SCC and Steel Fibers." Electron. J. Struct. Eng 14 (2014): 71-83.

Kaïkea, Adel, Djamel Achoura, François Duplan, and Lidia Rizzuti. "Effect of mineral admixtures and steel fiber volume contents on the behavior of high performance fiber reinforced concrete." Materials & Design 63 (2014): 493-499.

Tadepalli, Padmanabha Rao, Y. L. Mo, and Thomas TC Hsu. "Mechanical properties of steel fibre concrete." Magazine of Concrete Research 65, no. 8 (2013): 462-474.

Altun, Fatih, and Bekir Aktaş. "Investigation of reinforced concrete beams behavior of steel fiber added lightweight concrete." Construction and Building Materials 38 (2013): 575-581.

Germano, Federica, and Giovanni A. Plizzari. "Fatigue behavior of SFRC under bending." In Proceedings of the Eighth RILEM Intnl. Symp. on Fibre Reinforced Concrete: challenges and opportunities,(BEFIB 2012), pp. 19-21. 2012.

Randl, N., and F. Däuber. "Material properties of fibre reinforced UHPC." In 8th RILEM international symposium on fibre reinforced concrete: challenges and opportunities. PRO, vol. 88. 2012.

Sarmiento, E. V., G. Zirgulis, S. Sandbakk, M. R. Geiker, and T. Kanstad. "Influence of concrete flow on fibre distribution, orientation and mechanical properties of fibre reinforced concrete." In BEFIB2012—8th RILEM international symposium of fibre reinforced concrete, Guimaraes, Portugal, pp. 1-12. 2012.

Zerbino, Raúl L., and Bryan E. Barragán. "Long-Term Behavior of Cracked Steel Fiber-Reinforced Concrete Beams under Sustained Loading." ACI Materials Journal 109, no. 2 (2012).

Sharma, Satish, V. V. Arora, Suresh Kumar, Y. N. Daniel, and Ankit Sharma. "Durability Study of High-Strength Steel Fiber-Reinforced Concrete." ACI Materials Journal 115, no. 2 (2018): 219-225.

García-Taengua, Emilio, José R. Martí-Vargas, and Pedro Serna-Ros. "Statistical approach to effect of factors involved in bond performance of steel fiber-reinforced concrete." ACI Structural Journal 108, no. 4 (2011): 461-468.

Kelpša, Šarūnas, Mindaugas Augonis, Mindaugas Daukšys, and Algirdas Augonis. "Analysis of crack width calculation of steel fibre and ordinary reinforced concrete flexural members." Journal of Sustainable Architecture and Civil Engineering 6, no. 1 (2014): 50-57.

Cho, Hyun-Woo, Jae-Heum Moon, and Jang-Hwa Lee. "The Effects of Aggregate Sizes and Fiber Volume Fraction on Bending Toughness and Direct Tension of Steel Fiber Reinforced Concrete." World Academy of Science, Engineering and Technology, International Journal of Civil, Environmental, Structural, Construction and Architectural Engineering 6, no. 10 (2012): 816-820.

Iqbal, Shahid, Ahsan Ali, Klaus Holschemacher, and Thomas A. Bier. "Mechanical properties of steel fiber reinforced high strength lightweight self-compacting concrete (SHLSCC)." Construction and Building Materials 98 (2015): 325-333.

Wu, Zemei, Caijun Shi, Wen He, and Linmei Wu. "Effects of steel fiber content and shape on mechanical properties of ultra high performance concrete." Construction and building materials 103 (2016): 8-14.

Xie, Jian-he, Yong-chang Guo, Li-sha Liu, and Zhi-hong Xie. "Compressive and flexural behaviours of a new steel-fibre-reinforced recycled aggregate concrete with crumb rubber." Construction and Building materials 79 (2015): 263-272.

Kang, Su-Tae, and Jin-Keun Kim. "Investigation on the flexural behavior of UHPCC considering the effect of fiber orientation distribution." Construction and Building Materials 28, no. 1 (2012): 57-65.

Yoo, Doo-Yeol, Su-Tea Kang, and Young-Soo Yoon. "Effect of fiber length and placement method on flexural behavior, tension-softening curve, and fiber distribution characteristics of UHPFRC." Construction and Building materials 64 (2014): 67-81.

Kurihara, Norihiko, Minoru Kunieda, Toshiro Kamada, Yuichi Uchida, and Keitetsu Rokugo. "Tension softening diagrams and evaluation of properties of steel fiber reinforced concrete." Engineering Fracture Mechanics 65, no. 2-3 (2000): 235-245.

Lee, M. K., and B. I. G. Barr. "A four-exponential model to describe the behaviour of fibre reinforced concrete." Materials and structures 37, no. 7 (2004): 464-471.

Zhang, Jun, and Victor C. Li. "Simulation of crack propagation in fiber-reinforced concrete by fracture mechanics." Cement and Concrete Research 34, no. 2 (2004): 333-339.

Amirineni, Krishna C. "Fracture properties of fiber reinforced concrete." (2009).

Pereira, E. N. B., Joaquim AO Barros, Vitor MCF Cunha, and S. P. F. Santos. "Compression and bending behavior of steel fiber reinforced self-compacting concrete." (2005).

Barros, Joaquim AO, and José Sena-Cruz. "Fracture energy of steel fibre reinforced concrete." Journal of Mechanics of Composite Materials and Structures 8, no. 1 (2001): 29-45.

Soulioti, D., N. M. Barkoula, A. Paipetis, T. E. Matikas, T. Shiotani, and D. G. Aggelis. "Acoustic emission behavior of steel fibre reinforced concrete under bending." Construction and Building Materials 23, no. 12 (2009): 3532-3536.

Stähli, Patrick, Rocco Custer, and Jan GM van Mier. "On flow properties, fibre distribution, fibre orientation and flexural behaviour of FRC." Materials and Structures 41, no. 1 (2008): 189-196.

Giaccio, G., J. M. Tobes, and Raul Zerbino. "Use of small beams to obtain design parameters of fibre reinforced concrete." Cement and Concrete Composites 30, no. 4 (2008): 297-306.

Köksal, Fuat, Fatih Altun, İlhami Yiğit, and Yuşa Şahin. "Combined effect of silica fume and steel fiber on the mechanical properties of high strength concretes." Construction and building materials 22, no. 8 (2008): 1874-1880.

Pereira, E. N. B., Joaquim AO Barros, Alberto F. Ribeiro, and Aires Camões. "Post-cracking behaviour of selfcompacting steel fibre reinforced concrete." (2004).

Boulekbache, Bensaid, Mostefa Hamrat, Mohamed Chemrouk, and Sofiane Amziane. "Flowability of fibre-reinforced concrete and its effect on the mechanical properties of the material." Construction and Building Materials 24, no. 9 (2010): 1664-1671.

Jones, Peter A., Simon A. Austin, and Peter J. Robins. "Predicting the flexural load–deflection response of steel fibre reinforced concrete from strain, crack-width, fibre pull-out and distribution data." Materials and Structures 41, no. 3 (2008): 449-463.

Holschemacher, Klaus, and Torsten Müller. "Influence of fibre type on hardened properties of steel fibre reinforced concrete." Leipzig University of Applied Sciences (HTWK Leipzig), Departament of civil Enginieering (2007).

Barros, Joaquim AO, and J. A. B. Antunes. "Experimental characterization of the flexural behaviour of steel fibre reinforced concrete according to RILEM TC 162-TDF recommendations." RILEM TC 162 (2003): 77-89.

Vandewalle, Lucie, Gert Heirman, and Filip Van Rickstal. "Fibre orientation in self-compacting fibre reinforced concrete." In Proc. of the 7th Int. RILEM Symp. on Fibre Reinforced Concrete: Design and Applications (BEFIB2008), pp. 719-728. RILEM Publications SARL; Bagneux, 2008.

Concrete, Ultra High Performance, M. Schmidt, E. Fehling, and C. Geisenhansluke. "Ultra High Performance Concrete (UHPC)." (2004).

Choi, O. C., and C. Lee. "Flexural performance of ring-type steel fiber-reinforced concrete." Cement and concrete research 33, no. 6 (2003): 841-849.

Yoo, Doo-Yeol, Su-Tea Kang, Joo-Ha Lee, and Young-Soo Yoon. "Effect of shrinkage reducing admixture on tensile and flexural behaviors of UHPFRC considering fiber distribution characteristics." Cement and concrete research 54 (2013): 180-190.

Alani, Amir M., and Derrick Beckett. "Mechanical properties of a large scale synthetic fibre reinforced concrete ground slab." Construction and Building Materials 41 (2013): 335-344.

Lanzoni, Luca, Andrea Nobili, and Angelo Marcello Tarantino. "Performance evaluation of a polypropylene-based draw-wired fibre for concrete structures." Construction and building materials 28, no. 1 (2012): 798-806.

Soutsos, M. N., T. T. Le, and A. P. Lampropoulos. "Flexural performance of fibre reinforced concrete made with steel and synthetic fibres." Construction and building materials 36 (2012): 704-710.

Pujadas, Pablo, Ana Blanco, Sergio H. Cavalaro, Albert De la Fuente, and Antonio Aguado. "Flat suspended slabs reinforced only with macro-synthetic fibres." (2016).

Alberti, M. G., A. Enfedaque, J. C. Gálvez, and V. Agrawal. "Reliability of polyolefin fibre reinforced concrete beyond laboratory sizes and construction procedures." Composite Structures 140 (2016): 506-524.

Alberti, M. G., A. Enfedaque, and J. C. Gálvez. "Fracture mechanics of polyolefin fibre reinforced concrete: Study of the influence of the concrete properties, casting procedures, the fibre length and specimen size." Engineering Fracture Mechanics 154 (2016): 225-244.

Navas, F. Ortiz, Juan Navarro-Gregori, G. Leiva Herdocia, P. Serna, and E. Cuenca. "An experimental study on the shear behaviour of reinforced concrete beams with macro-synthetic fibres." Construction and Building Materials 169 (2018): 888-899.

Zerbino, R., Diego Hernán Monetti, and G. Giaccio. "Creep behaviour of cracked steel and macro-synthetic fibre reinforced concrete." Materials and Structures 49, no. 8 (2016): 3397-3410.

Noushini, Amin, Max Hastings, Arnaud Castel, and Farhad Aslani. "Mechanical and flexural performance of synthetic fibre reinforced geopolymer concrete." Construction and Building Materials 186 (2018): 454-475.

Kim, B., A. J. Boyd, H-S. Kim, and S-H. Lee. "Steel and synthetic types of fibre reinforced concrete exposed to chemical erosion." Construction and Building Materials 93 (2015): 720-728.

Babafemi, Adewumi John, and William Peter Boshoff. "Testing and modelling the creep of cracked macro-synthetic fibre reinforced concrete (MSFRC) under flexural loading." Materials and Structures 49, no. 10 (2016): 4389-4400.

Hardy, Nell, Stephen Foster, Ron Cox, Hamid Vali Pour Goudarzi, and Ali Amin. "Investigation into the use of macro synthetic fibre reinforced concrete for breakwater armour units." Coastal Engineering 140 (2018): 60-71.

Bester, Hermanus Lambertus. "Generic model for predicting the performance of macro-synthetic fibre reinforced concrete for industrial flooring applications." PhD diss., Stellenbosch: Stellenbosch University, 2017.

Alberti, M. G., A. Enfedaque, and J. C. Gálvez. "Comparison between polyolefin fibre reinforced vibrated conventional concrete and self-compacting concrete." Construction and Building Materials 85 (2015): 182-194.

Di Maida, Pietro, Corrado Sciancalepore, Enrico Radi, and Federica Bondioli. "Effects of nano-silica treatment on the flexural post cracking behaviour of polypropylene macro-synthetic fibre reinforced concrete." Mechanics Research Communications 88 (2018): 12-18.

Garcez, Estela O., Muhammad I. Kabir, Mahbube Subhani, Alastair MacLeod, Andras Fehervari, Mitchell Hall, and Patrick Moulton. "Development of high strength self-compacting fibre reinforced concrete for prefabricated concrete industry." In MATEC Web of Conferences, vol. 275, p. 02011. EDP Sciences, 2019.

Çelik, Zinnur, and Ahmet Ferhat Bingöl. "Effect of basalt, polypropylene and macro-synthetic fibres on workability and mechanical properties of self-compacting concrete." CHALLENGE 5, no. 2 (2019): 35-41.

Richardson, Alan, and Rhys Ovington. "Temperature related steel and synthetic fibre concrete performance." Construction and Building Materials 153 (2017): 616-621.

Hesami, Saeid, Iman Salehi Hikouei, and Seyed Amir Ali Emadi. "Mechanical behavior of self-compacting concrete pavements incorporating recycled tire rubber crumb and reinforced with polypropylene fiber." Journal of cleaner production 133 (2016): 228-234.

Afroughsabet, Vahid, and Togay Ozbakkaloglu. "Mechanical and durability properties of high-strength concrete containing steel and polypropylene fibers." Construction and building materials 94 (2015): 73-82.

Yew, Ming Kun, Hilmi Bin Mahmud, Bee Chin Ang, and Ming Chian Yew. "Influence of different types of polypropylene fibre on the mechanical properties of high-strength oil palm shell lightweight concrete." Construction and Building Materials 90 (2015): 36-43.

de la Fuente, Albert, Renata C. Escariz, Antonio D. de Figueiredo, and Antonio Aguado. "Design of macro-synthetic fibre reinforced concrete pipes." Construction and Building Materials 43 (2013): 523-532.

Simões, T., H. Costa, D. Dias-da-Costa, and E. N. B. S. Júlio. "Influence of fibres on the mechanical behaviour of fibre reinforced concrete matrixes." Construction and Building Materials 137 (2017): 548-556.

Conforti, Antonio, Giuseppe Tiberti, Giovanni A. Plizzari, Angelo Caratelli, and Alberto Meda. "Precast tunnel segments reinforced by macro-synthetic fibers." Tunnelling and Underground Space Technology 63 (2017): 1-11.

Pujadas, Pablo, Ana Blanco, Sergio Cavalaro, Albert de la Fuente, and Antonio Aguado. "Fibre distribution in macro-plastic fibre reinforced concrete slab-panels." Construction and building materials 64 (2014): 496-503.

Abaeian, Reza, Hamid Pesaran Behbahani, and Shahram Jalali Moslem. "Effects of high temperatures on mechanical behavior of high strength concrete reinforced with high performance synthetic macro polypropylene (HPP) fibres." Construction and Building Materials 165 (2018): 631-638.

Rooholamini, H., A. Hassani, and M. R. M. Aliha. "Evaluating the effect of macro-synthetic fibre on the mechanical properties of roller-compacted concrete pavement using response surface methodology." Construction and Building Materials 159 (2018): 517-529.

Bendjillali, K., M. Chemrouk, and B. Boulekbache. "Recycled synthetic waste fibres for the reinforcement of concrete." In WASTES–Solutions, Treatments and Opportunities II, pp. 9-15. CRC Press, 2017.

Richardson, Alan, and Kathryn Coventry. "Dovetailed and hybrid synthetic fibre concrete–impact, toughness and strength performance." Construction and Building Materials 78 (2015): 439-449.

Pešić, Ninoslav, Stana Živanović, Reyes Garcia, and Panos Papastergiou. "Mechanical properties of concrete reinforced with recycled HDPE plastic fibres." Construction and building materials 115 (2016): 362-370.

Reddy, K. Chiranjeevi, and Kolluru VL Subramaniam. "Analysis for multi-linear stress-crack opening cohesive relationship: Application to macro-synthetic fiber reinforced concrete." Engineering Fracture Mechanics 169 (2017): 128-145.

Juhász, Károly Péter, and Péter Schaul. "The effect of age and testing method on the added fracture energy of fibre reinforced concrete." Concrete Structures 20 (2019): 20-24.

Richardson, Alan, Kathryn Coventry, Thomas Lamb, and David Mackenzie. "The addition of synthetic fibres to concrete to improve impact/ballistic toughness." Construction and Building Materials 121 (2016): 612-621.

Lee, Su-Jin, Se-Ho Kim, and Jong-Pil Won. "Bond-flexural behaviour of structural nano-synthetic fibre-reinforced cementitious composites." Composite Structures 152 (2016): 20-33.

Borg, Ruben Paul, Owen Baldacchino, and Liberato Ferrara. "Early age performance and mechanical characteristics of recycled PET fibre reinforced concrete." Construction and Building Materials 108 (2016): 29-47.

Enfedaque, A., M. G. Alberti, J. A. Paredes, and J. C. Gálvez. "Interface properties of polyolefin fibres embedded in self-compacting concrete with a bond improver admixture." Theoretical and Applied Fracture Mechanics 90 (2017): 287-293.

Lerch, J. O., H. L. Bester, A. S. Van Rooyen, R. Combrinck, W. I. de Villiers, and W. P. Boshoff. "The effect of mixing on the performance of macro synthetic fibre reinforced concrete." Cement and Concrete Research 103 (2018): 130-139.

Di Maida, Pietro, Corrado Sciancalepore, Enrico Radi, and Federica Bondioli. "Effects of nano-silica treatment on the flexural post cracking behaviour of polypropylene macro-synthetic fibre reinforced concrete." Mechanics Research Communications 88 (2018): 12-18.

Bhosale, Aniket, M. Abdur Rasheed, S. Suriya Prakash, and Gangadharan Raju. "A study on the efficiency of steel vs. synthetic vs. hybrid fibers on fracture behavior of concrete in flexure using acoustic emission." Construction and Building Materials 199 (2019): 256-268.

Vandevyvere, Brecht, Zeger Sierens, Miquel Joseph, Paul Jonckheere, Luc Decraemer, and Jiabin Li. "Effect of PP fibres on flexural behaviour of concrete with RCAs–A preliminary study." In Proceedings of The 12th fib International PhD Symposium in Civil Engineering, pp. 1259-1265. Czech Technical University in Prague, 2018.

Yin, Shi, Rabin Tuladhar, Jacob Riella, David Chung, Tony Collister, Mark Combe, and Nagaratnam Sivakugan. "Comparative evaluation of virgin and recycled polypropylene fibre reinforced concrete." Construction and building materials 114 (2016): 134-141.

Kosior-Kazberuk, Marta. "Post-cracking Behaviour and Fracture Energy of Synthetic Fibre Reinforced Concrete." Materials Science 22, no. 4 (2016): 542-547.

Sahoo, Dipti Ranjan, Kaushik Maran, and Avdhesh Kumar. "Effect of steel and synthetic fibers on shear strength of RC beams without shear stirrups." Construction and Building Materials 83 (2015): 150-158.

de Souza Castoldi, Raylane, Lourdes Maria Silva de Souza, and Flávio de Andrade Silva. "Comparative study on the mechanical behavior and durability of polypropylene and sisal fiber reinforced concretes." Construction and Building Materials 211 (2019): 617-628.

Oliari Garcez, Estela, Muhammad Ikramul Kabir, Alastair MacLeod, Mahbube Subhani, and Kazem Ghabraie. "Self-Compacting Concrete Reinforced with Twisted-Bundle Macro-Synthetic Fiber." Applied Sciences 9, no. 12 (2019): 2543.

Hu, Hang, Panos Papastergiou, Harris Angelakopoulos, Maurizio Guadagnini, and Kypros Pilakoutas. "Mechanical properties of SFRC using blended manufactured and recycled tyre steel fibres." Construction and Building Materials 163 (2018): 376-389.

Sengün, E., Burhan Alam, and İ. Ö. Yaman. "Effect of synthetic fibers on flexural performance of normal and high performance concrete." In Proc., 9th RILEM Int. Symp. on Fiber Reinforced Concrete. 2016.

Çelik, Zinnur, and Ahmet Ferhat Bingöl. "Effect of basalt, polypropylene and macro-synthetic fibres on workability and mechanical properties of self-compacting concrete." CHALLENGE 5, no. 2 (2019): 35-41.

Amin, Ali, Stephen J. Foster, R. Ian Gilbert, and Walter Kaufmann. "Material characterisation of macro synthetic fibre reinforced concrete." Cement and Concrete Composites 84 (2017): 124-133.

Papayianni, I., and M. Papachristoforou. "Effect of high temperatures on steel fiber reinforced concrete with EAF slag aggregates."

Daneshfar, M., A. Hassani, M. R. M. Aliha, and F. Berto. "Evaluating Mechanical Properties of Macro-Synthetic Fiber-Reinforced Concrete with Various Types and Contents." Strength of Materials 49, no. 5 (2017): 618-626.

Yehia, Sherif, AlaEddin Douba, Omar Abdullahi, and Sharef Farrag. "Mechanical and durability evaluation of fiber-reinforced self-compacting concrete." Construction and Building Materials 121 (2016): 120-133.

Kazmi, Syed Minhaj Saleem, Muhammad Junaid Munir, Yu-Fei Wu, and Indubhushan Patnaikuni. "Effect of macro-synthetic fibers on the fracture energy and mechanical behavior of recycled aggregate concrete." Construction and Building Materials 189 (2018): 857-868.

Yazdanbakhsh, Ardavan, Salah Altoubat, and Klaus-Alexander Rieder. "Analytical study on shear strength of macro synthetic fiber reinforced concrete beams." Engineering Structures 100 (2015): 622-632.

Llano-Torre, Aitor, Emili García-Taengua, José R. Martí-Vargas, and Pedro Serna. "Compilation and study of a database of tests and results on flexural creep behavior of fibre reinforced concrete specimens." In FIB Symposium Proceedings. Leeds, 2015.

Rasheed, M. Abdur, S. Suriya Prakash, Gangadharan Raju, and Yuma Kawasaki. "Fracture studies on synthetic fiber reinforced cellular concrete using acoustic emission technique." Construction and Building Materials 169 (2018): 100-112.

Ige, Olubisi, S. J. Barnett, Ayman Nassif, and J. B. Williams. "Distribution and orientation of steel fibres in steel fibre reinforced concrete." In 4th International Conference on Advances in Civil, Structural and Construction Engineering, pp. 43-47. IRED, 2016.

Richardson, Alan, and David Batey. "Impact resistance of concrete using dovetailed fibres and type 2 synthetic fibres." (2015).

Mohod, Milind V. "Performance of polypropylene fibre reinforced concrete." IOSR Journal of Mechanical and Civil Engineering 12, no. 1 (2015): 28-36.

Dopko, Michael, Meysam Najimi, Behrouz Shafei, Xuhao Wang, Peter Taylor, and Brent M. Phares. "Flexural performance evaluation of fiber-reinforced concrete incorporating multiple macro-synthetic fibers." Transportation Research Record 2672, no. 27 (2018): 1-12.

Juhász, K. P., P. Schaul, and L. Nagy. "Effect of the loading rate on fibre reinforced concrete beams." In IOP Conference Series: Materials Science and Engineering, vol. 246, no. 1, p. 012040. IOP Publishing, 2017.

Ghaffar, Abdul, Amit S. Chavhan, and Dr RS Tatwawadi. "Steel Fibre reinforced concrete." International Journal of Engineering Trends and Technology (IJETT) 9, no. 15 (2014): 791-797.

Conforti, Antonio, Fausto Minelli, and Giovanni A. Plizzari. "Shear behaviour of prestressed double tees in self-compacting polypropylene fibre reinforced concrete." Engineering Structures 146 (2017): 93-104.

Zamanzadeh, Ziaaddin, Lúcio Lourenço, and Joaquim Barros. "Recycled steel fibre reinforced concrete failing in bending and in shear." Construction and Building Materials 85 (2015): 195-207.

Giaccio, G., M. E. Bossio, M. C. Torrijos, and R. Zerbino. "Contribution of fiber reinforcement in concrete affected by alkali–silica reaction." Cement and Concrete Research 67 (2015): 310-317.

Rana, Amit. "Some studies on steel fiber reinforced concrete." International journal of emerging technology and advanced engineering 3, no. 1 (2013): 120-127.

Pająk, M., and T. Ponikiewski. "Flexural behavior of self-compacting concrete reinforced with different types of steel fibers." Construction and Building Materials 47 (2013): 397-408.

Yin, Shi, Rabin Tuladhar, Tony Collister, Mark Combe, Nagaratnam Sivakugan, and Zongcai Deng. "Post-cracking performance of recycled polypropylene fibre in concrete." Construction and Building Materials 101 (2015): 1069-1077.

Sukontasukkul, Piti, and Pitthaya Jamsawang. "Use of steel and polypropylene fibers to improve flexural performance of deep soil–cement column." Construction and Building Materials 29 (2012): 201-205.

Pelisser, Fernando, Oscar Rubem Klegues Montedo, Philippe Jean Paul Gleize, and Humberto Ramos Roman. "Mechanical properties of recycled PET fibers in concrete." Materials research 15, no. 4 (2012): 679-686.

Lee, Su-Jin, and Jong-Pil Won. "Flexural behavior of precast reinforced concrete composite members reinforced with structural nano-synthetic and steel fibers." Composite Structures 118 (2014): 571-579.

Babafemi, Adewumi John. "Tensile creep of cracked macro synthetic fibre reinforced concrete." PhD diss., Stellenbosch: Stellenbosch University, 2015.

Alani, Amir, Morteza Aboutalebi, and Martin J. King. "Influence of fibre content on crack propagation rate in fibre-reinforced concrete beams." International Journal of Civil, Environmental, Structural, Construction and Architectural Engineering 7, no. 9 (2013): 1-7.

Buratti, N., and C. Mazzotti. "Experimental tests on the effect of temperature on the long-term behaviour of macrosynthetic Fibre Reinforced Concretes." Construction and Building Materials 95 (2015): 133-142.

Conforti, Antonio, Raúl Zerbino, and Giovanni A. Plizzari. "Influence of steel, glass and polymer fibers on the cracking behavior of reinforced concrete beams under flexure." Structural Concrete 20, no. 1 (2019): 133-143.

Picazo, Álvaro, Marcos G. Alberti, Jaime C. Gálvez, Alejandro Enfedaque, and Abner C. Vega. "The size effect on flexural fracture of polyolefin fibre-reinforced concrete." Applied Sciences 9, no. 9 (2019): 1762.

de Rivaz, Benoit. "Fibre reinforced spray concrete for compliance with site safety requirement." Concreto y cemento. Investigación y desarrollo 2, no. 2 (2011): 48-58.

Liu, Xingzi, Mengpei Yan, Isaac Galobardes, and Karol Sikora. "Assessing the potential of functionally graded concrete using fibre reinforced and recycled aggregate concrete." Construction and Building Materials 171 (2018): 793-801.

Domski, Jacek, and Jacek Katzer. "Load-deflection characteristic of fibre concrete based on waste ceramic aggregate." Annual Set The Environment Protection 15 (2013): 213-230.

Monetti, Diego Hernán, A. Llano-Torre, María Celeste Torrijos, G. Giaccio, Raúl Zerbino, J. R. Martí-Vargas, and P. Serna. "Long-term behavior of cracked fiber reinforced concrete under service conditions." Construction and Building Materials 196 (2019): 649-658.

Ponikiewski, Tomasz, and Jacek Katzer. "X-ray computed tomography of fibre reinforced self-compacting concrete as a tool of assessing its flexural behaviour." Materials and Structures 49, no. 6 (2016): 2131-2140.

Singh, S. P., A. P. Singh, and V. Bajaj. "Strength and flexural toughness of concrete reinforced with steel-polypropylene hybrid fibres." (2010): 495-507.

Conforti, Antonio, Fausto Minelli, Giovanni A. Plizzari, and Giuseppe Tiberti. "Comparing test methods for the mechanical characterization of fiber reinforced concrete." Structural Concrete 19, no. 3 (2018): 656-669.

Shinde, Pravin B., Sangita V. Pawar, and V. P. Kulkarni. "Flexural behaviour of hybrid fiber reinforced concrete deep beam and effect of steel polypropylene fiber on mechanical properties of concrete." Int. J. Adv. Res. Sci. Eng 4, no. 2 (2015): 62-73.

Chiranjeevi Reddy, Kamasani, and Kolluru VL Subramaniam. "Experimental investigation of crack propagation and post-cracking behaviour in macrosynthetic fibre reinforced concrete." Magazine of Concrete Research 69, no. 9 (2017): 467-478.

Llano-Torre, Aitor, Samuel Eduardo Arango, Emilio García-Taengua, José Rocío Martí-Vargas, and Pedro Serna. "Influence of fibre reinforcement on the long-term behaviour of cracked concrete." In Creep Behaviour in Cracked Sections of Fibre Reinforced Concrete, pp. 195-209. Springer, Dordrecht, 2017.

Conforti, A., G. A. Plizzari, and R. Zerbino. "Vibrated and self-compacting fibre reinforced concrete: experimental investigation on the fibre orientation." In IOP Conference Series: Materials Science and Engineering, vol. 246, no. 1, p. 012019. IOP Publishing, 2017.

de Rivaz, B., and Y. Ding. "Relevant Perform ance Characterisation Test for Fibre Reinforced Sprayed Concrete."

Dawood, Eethar Thanon, and Mahyuddin Ramli. "High strength characteristics of cement mortar reinforced with hybrid fibres." Construction and building materials 25, no. 5 (2011): 2240-2247.

Stephen, J. S., Gettu, R., and R. Raphael. "Effect of loading rate on the fracture behavior of fibre reinforced concrete." In 9th international conference on fracture mechanics of concrete and concrete structures, Proceedings FraMCoS-9. https://doi. org/10.21012/FC9, vol. 71. 2016.

Choumanidis, D., E. Badogiannis, P. Nomikos, and A. Sofianos. "The effect of different fibres on the flexural behaviour of concrete exposed to normal and elevated temperatures." Construction and Building Materials 129 (2016): 266-277.

Noushini, Amin, Kirk Vessalas, and Bijan Samali. "Static mechanical properties of polyvinyl alcohol fibre reinforced concrete (PVA-FRC)." Magazine of Concrete Research 66, no. 9 (2014): 465-483.

Richardson, Alan, and Rhys Ovington. "Performance of fibre concrete with regard to temperature." (2017): 63-63.

Conforti, Antonio, Fausto Minelli, Andrea Tinini, and Giovanni A. Plizzari. "Influence of polypropylene fibre reinforcement and width-to-effective depth ratio in wide-shallow beams." Engineering Structures 88 (2015): 12-21.

Alberti, Marcos G., Alejandro Enfedaque, Jaime C. Gálvez, and Luis Pinillos. "Structural Cast-in-Place Application of Polyolefin Fiber–Reinforced Concrete in a Water Pipeline Supporting Elements." Journal of Pipeline Systems Engineering and Practice 8, no. 4 (2017): 05017002.

Vrijdaghs, Rutger, Marco di Prisco, and L. U. C. I. E. Vandewalle. "Creep of cracked polymer fiber reinforced concrete under sustained tensile loading." In FRAMCOS 9, pp. 1-9. University of California, Berkeley Clark Kerr Campus, 2016.

Yu, R., P. Spiesz, and H. J. H. Brouwers. "Development of Ultra-High Performance Fibre Reinforced Concrete (UHPFRC): Towards an efficient utilization of binders and fibres." Construction and building materials 79 (2015): 273-282.

Won, Jong-Pil, Byung-Tak Hong, Tei-Joon Choi, Su-Jin Lee, and Joo-Won Kang. "Flexural behaviour of amorphous micro-steel fibre-reinforced cement composites." Composite Structures 94, no. 4 (2012): 1443-1449.

López-Buendía, Angel M., María Dolores Romero-Sánchez, Verónica Climent, and Celia Guillem. "Surface treated polypropylene (PP) fibres for reinforced concrete." Cement and Concrete Research 54 (2013): 29-35.

Shende, A. M., A. M. Pande, and M. Gulfam Pathan. "Experimental study on steel fiber reinforced concrete for M-40 grade." International Refereed Journal of Engineering and Science 1, no. 1 (2012): 043-048.

Mohod, Milind V. "Performance of steel fiber reinforced concrete." International Journal of Engineering and Science 1, no. 12 (2012): 1-4.

Meda, Alberto, Fausto Minelli, and Giovanni A. Plizzari. "Flexural behaviour of RC beams in fibre reinforced concrete." Composites Part B: Engineering 43, no. 8 (2012): 2930-2937.

Zerbino, R., J. M. Tobes, M. E. Bossio, and G. Giaccio. "On the orientation of fibres in structural members fabricated with self compacting fibre reinforced concrete." Cement and Concrete Composites 34, no. 2 (2012): 191-200.

Alberti, Marcos G., Alejandro Enfedaque, Jaime C. Gálvez, and Carlos Álvarez. "Using Polyolefin Fibers with Moderate-Strength Concrete Matrix to Improve Ductility." Journal of Materials in Civil Engineering 31, no. 9 (2019): 04019170.

Badogiannis, E. G., K. I. Christidis, and G. E. Tzanetatos. "Evaluation of the mechanical behavior of pumice lightweight concrete reinforced with steel and polypropylene fibers." Construction and Building Materials 196 (2019): 443-456.

Gali, Sahith, and Kolluru VL Subramaniam. "Multi-linear stress-crack separation relationship for steel fiber reinforced concrete: Analytical framework and experimental evaluation." Theoretical and Applied Fracture Mechanics 93 (2018): 33-43.

Galeote, Eduardo, Ana Blanco, Sergio HP Cavalaro, and Albert De la Fuente. "Correlation between the Barcelona test and the bending test in fibre reinforced concrete." Construction and Building Materials 152 (2017): 529-538.

Ortega-López, V., V. Revilla-Cuesta, M. Skaf, F. Fiol, A. Santamaría, A. García-Llona, and I. Piñero. "Fracture Toughness Evaluation Of Fiber-Reinforced Concrete Manufactured With Siderurgic Aggregates."

Conforti, Antonio, Giuseppe Tiberti, and Giovanni A. Plizzari. "Combined effect of high concentrated loads exerted by TBM hydraulic jacks." Magazine of Concrete Research 68, no. 21 (2016): 1122-1132.

Sandbakk, Sindre. "Fibre Reinforced Concrete: Evaluation of test methods and material development." (2011).

Lee, Su-Jin, Yerin Hong, Ah-Hyeon Eom, and Jong-Pil Won. "Effect of steel fibres on fracture parameters of cementitious composites." Composite Structures 204 (2018): 658-663.

Buratti, Nicola, Claudio Mazzotti, and Marco Savoia. "Identification of Constitutive Relationships for Fibre Reinforced Concretes by Inverse Analysis."

de Alencar Monteiro, Vitor Moreira, and Flávio de Andrade Silva. "Mechanical Behaviour Of Polypropylene And Steel Fiber Self-Consolidating Concrete."

Alberti, M. G., A. Enfedaque, J. C. Gálvez, and A. Cortez. "Optimisation of fibre reinforcement with a combination strategy and through the use of self-compacting concrete." Construction and Building Materials 235 (2020): 117289.

Tiberti, Giuseppe, Antonio Conforti, and Giovanni A. Plizzari. "Precast segments under TBM hydraulic jacks: Experimental investigation on the local splitting behavior." Tunnelling and Underground Space Technology 50 (2015): 438-450.

Isla, F., B. Luccioni, G. Ruano, M. C. Torrijos, F. Morea, G. Giaccio, and R. Zerbino. "Mechanical response of fiber reinforced concrete overlays over asphalt concrete substrate: experimental results and numerical simulation." Construction and Building Materials 93 (2015): 1022-1033.

Boulekbache, Bensaid, Mostefa Hamrat, Mohamed Chemrouk, and Sofiane Amziane. "Flowability of fibre-reinforced concrete and its effect on the mechanical properties of the material." Construction and Building Materials 24, no. 9 (2010): 1664-1671.

Lameiras, Rodrigo, Joaquim Barros, Isabel B. Valente, and Miguel Azenha. "Development of sandwich panels combining fibre reinforced concrete layers and fibre reinforced polymer connectors. Part I: Conception and pull-out tests." Composite Structures 105 (2013): 446-459.

Yang, In-Hwan, Changbin Joh, and Byung-Suk Kim. "Flexural response predictions for ultra-high-performance fibre-reinforced concrete beams." Magazine of Concrete Research 64, no. 2 (2012): 113-127.

Kamasani, Chiranjeevi Reddy, and Kolluru VL Subramaniam. "Experimental evaluation of flexural response and postcracking behavior in macro-synthetic fiber reinforced concrete." (2016): 64-74.

Park, Kyoungsoo, Glaucio H. Paulino, and Jeffery Roesler. "Cohesive fracture model for functionally graded fiber reinforced concrete." Cement and concrete research 40, no. 6 (2010): 956-965.

Richardson, A. E., and P. Jackson. "Equating steel and synthetic fibre concrete post crack performance." In 2nd International Conference on Current Trends in Technology, NUiCONE, December 8–10. Nirma University Ahmedabad, India, 2011.

Parmentier, Benoit, Niki Cauberg, and Lucie Vandewalle. "Shear resistance of macro-synthetic and steel fibre reinforced concrete beams without stirrups." In 8th RILEM International Symposium on Fiber Reinforced Concrete: Challenges and Opportunities. 2012.

Buratti, Nicola, Claudio Mazzotti, and Marco Savoia. "Post-cracking behaviour of steel and macro-synthetic fibre-reinforced concretes." Construction and Building Materials 25, no. 5 (2011): 2713-2722.

Noushini, Amin, Bijan Samali, and Kirk Vessalas. "Flexural toughness and ductility characteristics of polyvinyl-alcohol fibre reinforced concrete (PVA-FRC)." In Proceedings of the 8th International Conference on Fracture Mechanics of Concrete and Concrete Structures, FraMCoS 2013. 2013.

Mobasher, Barzin, Mehdi Bakhshi, and Christopher Barsby. "Backcalculation of residual tensile strength of regular and high performance fiber reinforced concrete from flexural tests." Construction and Building Materials 70 (2014): 243-253.

Erdem, Savaş, Andrew Robert Dawson, and Nicholas Howard Thom. "Microstructure-linked strength properties and impact response of conventional and recycled concrete reinforced with steel and synthetic macro fibres." Construction and Building Materials 25, no. 10 (2011): 4025-4036.

Alberti, M. G., A. Enfedaque, and J. C. Gálvez. "On the mechanical properties and fracture behavior of polyolefin fiber-reinforced self-compacting concrete." Construction and building materials 55 (2014): 274-288.

Denneman, Erik, Rongzong Wu, Elsabe P. Kearsley, and Alex T. Visser. "Discrete fracture in high performance fibre reinforced concrete materials." Engineering Fracture Mechanics 78, no. 10 (2011): 2235-2245.

Jose, Sujatha, S. J. Stephan, and Ravindra Gettu. "Study of the post-cracking behavior of steel and polymer fibre reinforced concretes." 2nd RN Raikar Memorial Biennial International Conferences & 'Banthia–Basheer International Symposium. Mumbai, India, 2015.

Aslani, Farhad, and Morteza Bastami. "Relationship between deflection and crack mouth opening displacement of self-compacting concrete beams with and without fibers." Mechanics of Advanced Materials and Structures 22, no. 11 (2015): 956-967.

Yin, Shi, Rabin Tuladhar, Tony Collister, Mark Combe, Nagaratnam Sivakugan, and Zongcai Deng. "Post-cracking performance of recycled polypropylene fibre in concrete." Construction and building materials 101 (2015): 1069-1077.

Alberti, M. G., A. Enfedaque, and J. C. Gálvez. "Comparison between polyolefin fibre reinforced vibrated conventional concrete and self-compacting concrete." Construction and building materials 85 (2015): 182-194.

Buratti, N., C. Mazzotti, and M. Savoia. "Experimental study on the flexural behaviour of fibre reinforced concretes strengthened with steel and macro-synthetic fibres." Fracture Mechanics of Concrete and Concrete Structures-Assessment, Proceedings of FraMCoS-7, May (2010): 23-28.

Noushini, Amin, Kirk Vessalas, Nassim Ghosni, and Bijan Samali. "Effect of polyvinyl alcohol fibre and fly ash on flexural tensile properties of concrete." In From Materials to Structures: Advancement Through Innovation-Proceedings of the 22nd Australasian Conference on the Mechanics of Structures and Materials, pp. 1165-1170. 2013.

Wang, Jun-Yan, Nemkumar Banthia, and Min-Hong Zhang. "Effect of shrinkage reducing admixture on flexural behaviors of fiber reinforced cementitious composites." Cement and Concrete Composites 34, no. 4 (2012): 443-450.

Alani, Amir, and Morteza Aboutalebi. "Mechanical properties of fibre reinforced concrete-a comparative experimental study." International Journal of Civil, Environmental, Structural, Construction and Architectural Engineering 7, no. 9 (2013): 646-651.
